# Rare disease patients protection: theoretical discussion and institutional construction of orphan drug designation in China

**DOI:** 10.1186/s13023-026-04404-4

**Published:** 2026-05-25

**Authors:** Xiaopei Zhao, Mengyao Zhang

**Affiliations:** 1 School of Medical Humanities, Capital Medical University, NO.10, Xitoutiao, Outside You’an Gate, Beijing, 100069 China; 2 Department of Education, Cancer Hospital Chinese Academy of Medical Sciences, Beijing, 100000 China

**Keywords:** Rare disease patients, Orphan drug, Designation, Epidemiological criteria

## Abstract

Orphan drugs play a vital role, often described as the “public goods of public goods.” As a result, they demand robust policy support. Among these, the designation of orphan drugs serves as the pivotal and core element of the national incentive mechanism for such drugs. The designation of orphan drugs is a prerequisite and a key system to determine whether pharmaceutical enterprises are eligible to access policy support, which directly determines the effective output of orphan drugs and the fairness of public policies. However, there is still an urgent need to explore this system in China theoretically, and there is still a gap in legislation. This is an important root cause of the insufficient supply of orphan drugs in China. Therefore, on the premise of understanding its nature, and on the basis of reasonable reference to the existing legislation in overseas countries, and based on China’s realities and legislative framework, we should construct and improve China’s orphan drug designation system as soon as possible, so as to increase the rate of R&D and marketing of orphan drugs, enhance the level of treatment for patients with rare diseases, and help the smooth realization of the “Healthy China Strategy”.

## Introduction

Rare disease is a general term for diseases with extremely low incidence or prevalence rates, and extremely high disability and mortality rates. The World Health Organization (WHO) typically defines a single disease with a prevalence rate of less than 0.65‰-1‰ of the total population as a rare disease [1]. Different countries and regions define rare diseases differently based on their own medical conditions, population, and economic levels. Currently, Orphanet, the largest rare disease database in the world, has collected 6172 unique rare diseases, of which 71.9% are hereditary and 69.9% are childhood-onset diseases, and it is conservatively estimated that the global population affected by rare diseases is 263–446 million [2]. China is a country with a relatively large number of rare disease patients, and the Joint Meeting of Chairpersons of National Rare Disease Academic Groups has released a report stating that there are currently more than 1,400 types of rare diseases in China, with about 20 million patients with rare diseases [3]. Enhancing the accessibility of drugs and the level of treatment for patients with rare diseases is a yardstick of social civilization and is crucial to the foundation of people’s well-being.

Due to the low prevalence of individual rare diseases and the diverse etiologies, the development of drugs for rare diseases is complex, with high research and development (R&D) costs and minimal returns. As rational entities in the market economy, enterprises lack enthusiasm for R&D of drugs for rare diseases. Therefore, rare diseases are commonly referred to as “orphan diseases” and the medicines for them are called “orphan drugs” [4]. The field of rare disease drugs suffers from serious market failure. Without government intervention in the pharmaceutical market, patients with rare diseases would gradually become a marginalized group.

To promote R&D and marketing of rare disease drugs, the United States passed the Orphan Drug Act in 1983, pioneering the Orphan Drug Designation system [5]. This system has revolutionized the innovation of orphan drugs by linking designation with incentive measures to form a national incentive framework for rare disease drugs. Since then, many countries or regions around the world, such as Singapore (1991), Japan (1993), Australia (1997), the European Union (2000), and South Korea (2016), have successively enacted legislation to regulate the designation system for orphan drugs [6, 7]. Orphan drug designation is a prerequisite for enterprises to enjoy external incentive resources and promote iteration and innovation of medicines; it is also the core of the government’s incentive system for orphan drugs. By using designation as a tool, the government can accurately screen out medicines with clinical value and R&D potential, and efficiently allocates limited public resources through incentives.

The Chinese government has always attached great importance to ensuring the accessibility of medicines for patients with rare diseases. For example, the Drug Administration Law of the People’s Republic of China, amended in 2019, proposed the establishment of a national monitoring system for drug supply and demand, with early warnings for drugs in short supply. This regulation reveals that the state places great emphasis on rare disease drugs and other easily deficient drugs. The Law of the People’s Republic of China on Basic Medical and Health Care and the Promotion of Health, which came into force in June 2020, explicitly supports R&D of rare diseases drugs to meet the needs of disease prevention and treatment. The Regulations on the Implementation of the Drug Administration Law of the People’s Republic of China shall enter into force on May 15, 2026, of which Article 21 stipulates that “the state supports the research, development and innovation of medicines for the treatment of rare diseases. " However, the designation of orphan drugs in China has long lacked clear institutional provisions. In practice, rare diseases are mainly defined through two batches of “Rare Disease Catalogs” jointly issued by the National Health Commission and other departments in 2018 and 2023 respectively, and then the scope of drugs R&D is delimited. There are only 207 rare diseases in the catalogs, which is a limited number of categories. It is difficult for enterprises to accurately predict the direction of drug R&D. Coupled with the uncertainties in the market approval, market prospects, and returns of orphan drugs, the R&D motivation of enterprises is severely insufficient. In the long run, it has been unfavorable for the construction of an independent rare diseases drugs marketing system in China. The designation of orphan drugs requires systematic and scientific criteria and institutional provisions. To promote the rapid R&D and marketing of orphan drugs in China, it is extremely urgent to build a Chinese orphan drug designation system based on reasonable reference to existing foreign systems and full consideration of the national legislative framework.

This study fills the gap in systematic social science research on the qualification determination system for rare disease drugs in China. Its conclusions can not only directly support the ongoing legislative work related to rare diseases and rare disease drugs in China, but also provide reference experiences for similar legislative practices in other developing countries.

## The dilemmas in establishment of the designation system for orphan drugs

At the macro level, the orphan drug designation optimizes the distribution of national healthcare resources, so that patients with rare diseases are no longer marginalised in terms of access to healthcare resources, and improves the fairness and integrity of the entire healthcare system. However, as a matter of balance and equity in national public policy, the system is controversial and subject to the following conceptual constraints:

### Minority benefits

Although from an ethical and moral perspective, it is a recognized governmental and social responsibility of contemporary societies to address the unmet health needs of patients and to develop innovative treatments for them, reflecting the concepts of humanitarian care and the primacy of life. Contrary to this, however, orphan drugs face the dilemma of balancing cost-effectiveness. Under the constraints of limited public resources, it is difficult to achieve optimal allocation of resources by favouring a small number of patients with rare diseases. In particular, tax breaks to promote orphan drugs innovation will result in direct financial losses [8]. After all, there are only a handful of people with each rare disease, and it is against the principle of utilitarianism to invest a large amount of resources in a small group of rare patients.

With the in-depth research into diseases, the number of rare disease types and the number of patients are increasing year by year, and the treatment costs are also rising continuously. In the U.S., the per capita cost of rare disease patients treated in 2019 was $32,000, with treatments ranging from $6,000 to $500,000, and for orphan drugs priced at $100,000 per year, it could reach $1 billion per year if only 10,000 patients are treated [9]. Moreover, most orphan drugs have high list prices, with 39 per cent costing more than $100,000 per year [10]. This poses a threat to insurance funds, which in turn affects the level of coverage for other individual medicines. As the number of orphan drugs entering the market continues to increase, the impact is also exacerbated. This means that investing more in the R&D of orphan drugs will have an impact on the level of healthcare coverage for others, such as those with serious conditions such as chronic illnesses, which also require large amounts of funding for medical research to continually search for new therapies.

### Enterprises abuse the incentive measures for designation

There may be inappropriate use of incentives by enterprises in the orphan drug designation, raising a range of fairness issues. First is the high pricing behavior of orphan drugs. For example, in the United States, orphan drugs are approximately five times more expensive than non-orphan drugs ($150,854 versus $33,654 per year) [11]. Some orphan drugs have received multiple designations for treating multiple indications. This is supposed to promote the innovative use of drugs and the optimization of healthcare resources. However, the potential adverse consequence is that after the drug is launched, due to its multiple indications, the market demand increases. In response, enterprises may take the opportunity to raise the market price of the drug. The practice of enterprises enjoying multiple incentives and raising the market price of drugs at the same time breaks the original purpose of the incentive policy, which is to balance the public interest and corporate benefits, and goes against the government’s goal of improving the accessibility of drugs to patients with rare diseases through the incentive mechanism. Moreover, orphan drugs are inherently developed for diseases that lack effective treatment options, leaving patients with no choice but to use these high-priced medications. Furthermore, patient advocacy organizations such as the National Organization for Rare Disorders (NORD) in the United States and the European Organisation for Rare Diseases (EURORDIS) in the European Union show compassion and support for patients. As a result, the high pricing of orphan drugs seems to be reluctantly tolerated in practice [8]. The high pricing phenomenon of orphan drugs channels more medical funds towards pharmaceutical companies. This, in turn, can impact the overall allocation of medical resources in a country and the incentive mechanisms for orphan drugs development, ultimately harming patients with rare diseases.

Second is the monopolistic behavior of pharmaceuticals. After orphan drugs are designated and approved for marketing, they are granted a market exclusivity period of 7 or 10 years in various countries, which is much longer than the 5 to 6 years of exclusivity typically enjoyed by other pharmaceuticals. This gives them a solid position in the market. With the advancement of technology, enterprises can obtain multiple approvals for existing diseases or subtypes of indications, thereby continuously gaining additional periods of market exclusivity. This practice is known as “salami slicing” [8]. Given the high R&D costs of orphan drugs and the low number of potential patients, orphan drugs are not sold in large quantities in the conventional pharmaceutical market. Even when the drug exclusivity period expires, other enterprises are unlikely to rush to invest in the development of orphan drug generics. This effectively creates a quasi-monopoly position for orphan drugs in the market [8]. In the absence of competition, manufacturers can still make a profit over an extended monopoly period after incurring high costs to develop drugs for very rare diseases. This situation restricts the adjustability of drug prices, potentially leaving patients in a long-term predicament of having to pay high prices for medications. Moreover, it hinders the pharmaceutical industry from achieving technological innovation and cost optimization through robust competition, which is detrimental to the rational allocation and efficient use of medical resources on a broader scale.

## The solutions to the dilemmas in orphan drug designation

It is essential to establish the correct values for the designation of orphan drugs. The Declaration of the Ninth Global Conference on Health Promotion by WHO states, “We reaffirm our obligation to invest in health, to ensure universal health coverage, and to reduce health inequities for people of all ages. We are determined to do ‘no one less’” (The Shanghai Declaration on Health Promotion in the 2030 Agenda for Sustainable Development, Ninth Global Conference on Health Promotion, 2017) [12]. Civilized societies have a moral obligation to help minorities with serious diseases. Orphan drug designation is of great significance to patients with rare diseases. Since the enactment of the Orphan Drug Act in the United States in 1983 and up to 31 December 2022, a total of 6,340 orphan drugs have been designated, representing 1,079 diseases, of which 1,122 have received marketing approval [13]. The number of orphan drugs designated by the European Committee for Orphan Medicinal Products (COMP) grew by an average of 10% per year between 2011 and 2020 [14]. For example, in April 2018, the U.S. Food and Drug Administration (FDA) designated and approved Crysvita (generic name: burosumab-twza), the world’s first targeted therapy for X-linked hypophosphatemia (XLH), for patients aged 1 year and older [15]. As a rare inherited form of rickets, XLH had long lacked effective treatments addressing the underlying cause prior to Crysvita’s approval, leaving patients to suffer chronically from skeletal deformities, joint pain, limited mobility, and other debilitating symptoms. Crysvita serves as a prime example of how Orphan Drug Designation accelerates the availability of rare disease therapeutics [16], amply demonstrating that this system can translate the urgent medical needs of rare disease populations into accessible healthcare resources, thereby effectively safeguarding patients’ rights to health.

However, it is crucial to emphasize that the designation of orphan drugs must be scientific and prudent. This is related to the protection of the health rights of patients with rare diseases, the fair and rational allocation of medical resources, and the sustainable development of the pharmaceutical industry. Contemporary civilized society certainly has an inescapable moral and legal responsibility to patients with rare diseases. However, if we only rhetorically declare an inescapable moral obligation to the rare disease community in ethics or policy-making without considering, calculating, and balancing the actual economic burden, then humanitarianism will only become a slogan and a formality [17]. Therefore, based on the following principles, taking into full consideration the economic capacity of the country is a necessary prerequisite for achieving true humanitarian care:

### Principle of protection of vulnerable groups

From the perspective of governmental responsibility, it is the duty of the government to promote R&D of orphan drugs in contemporary society. Rare disease patients, as a socially disadvantaged group, have been facing the plight of disease suffering and lack of medicines for a long period of time. Satisfying their and their families’ urgent needs for therapeutic medicines is undoubtedly an important manifestation of the government’s positive response to the needs of the public. From a political point of view, if the survival and physical and mental health of vulnerable groups are not guaranteed for a long period of time, it may trigger social group conflicts and an increase in the crime rate, which are issues related to social stability [18]. Orphan drugs are essentially a “public product within a public product” [19]. The strong promotion and incentives from the government are particularly crucial. By implementing effective incentive measures and strict regulatory means, the government can accelerate the innovation of orphan drugs and effectively safeguard the rights and interests of patients with rare diseases.

From an ethical perspective, ethical theories provide a moral foundation for the protection of vulnerable groups. That is, protecting the weak is a defence of human dignity, which can promote the overall well-being of society and contribute to the cultivation of the common good [20]. Due to their inherent limitations, vulnerable groups often find themselves marginalized in society. Providing effective protection for their rights and interests demonstrates a civilized value system that respects every individual. John Rawls, a prominent philosopher, advocated for the theory of distributive justice, focusing on the protection of vulnerable groups. He proposed the principle of difference, which states that social and economic inequalities should be arranged so that they are to the greatest benefit of the least advantaged members of society [21]. Rare disease patients undoubtedly belong to the disadvantaged social groups. According to Rawls’ principle of difference, the government and society should take positive and effective measures to maximise their welfare.

At the institutional level, the issue of the accessibility of medical services and medications for rare - disease patients is particularly prominent. This highlights the urgency and significance of continuously strengthening the construction of relevant systems and enhancing the level of accessibility. The purpose of orphan drug legislation should be to resolve the problem of drug accessibility and stimulate R&D enthusiasm of enterprises. For instance, EU Regulation (EC) No 141/2000 explicitly states that the regulation aims at addressing the medical inequality faced by patients with rare diseases and encouraging pharmaceutical companies to engage in R&D of orphan drugs by providing incentives [22]. During the legislative process, it is necessary to clarify the criteria for orphan drug recognition and formulate a series of incentive mechanisms for enterprises, which will provide strong institutional support for safeguarding the rights and interests of patients with rare diseases.

### Spillover effects

The pathogenesis of rare diseases is often highly complex, not only manifesting as diverse genetic mutations but also involving multiple factors such as abnormalities in cellular signaling pathways, changes in protein function, and dysregulation of the immune system. With the continuous in-depth research by modern medical science and technology into human pathogenesis, traditional medical technologies are gradually showing their limitations in tackling rare diseases and other complex conditions. In the process of drug R&D, enterprises have to explore new technological pathways, tap into cutting-edge therapeutic targets, and try innovative drug dosage forms and delivery methods. Advanced Therapy Medicinal Products (ATMPs), centered on gene therapy and somatic cell therapy, have attracted great attention and emerged as a strategic high ground in global rare disease drug R&D. Take Casgevy, the world’s first approved CRISPR-Cas9 gene editing therapy, as an example. This drug has been approved for marketing in the UK, the US, and the EU successively, indicated for the treatment of sickle cell disease and transfusion-dependent β-thalassemia—both rare hematologic disorders caused by hemoglobin gene mutations [23]. Casgevy uses the innovative CRISPR-Cas9 gene editing tool to precisely cut and edit defective genes in patients’ bone marrow stem cells, enabling the body to produce functionally normal hemoglobin. However, the global supply of such innovative drugs is marked by prominent regional disparities. From the perspective of the global R&D landscape, China has accumulated solid technical reserves in the field of ATMPs. For the indication of transfusion-dependent β-thalassemia, the CRISPR gene editing therapy developed by Chinese biopharmaceutical firm EdiGene completed its Phase I clinical trial by the end of 2022 [24]. Yet constrained by factors including the ongoing optimization of the regulatory review system for cutting-edge therapies and the high difficulty of patient recruitment for rare disease clinical trials, its subsequent development and marketing application process has been slow, which further highlights the severe challenges facing the accessibility of rare disease treatments. Therefore, rare disease drug R&D is not only a frontier field of scientific exploration, but also a core benchmark for measuring medical equity. To effectively translate scientific breakthroughs into tangible and accessible health benefits, it is imperative to facilitate collaborative efforts among governments, pharmaceutical enterprises and various social stakeholders, to ensure that innovative outcomes ultimately benefit the broad patient community. The new knowledge, technologies and methods accumulated during these medical explorations have not only directly driven the innovation and development of orphan drugs, but also provided valuable reference for drug development for other common diseases.

Moreover, the phenomenon of cross-indication R&D in pharmaceuticals is increasingly common. This cross-boundary application of results has brought about groundbreaking changes and far-reaching impacts to the entire field of medical science. Drugs originally developed for the treatment of rare diseases have been proven to be significantly effective for other conditions in subsequent clinical practice and research. For example, Rituximab, which was initially designated as an orphan drug by the US FDA and approved for the treatment of follicular non-Hodgkin’s lymphoma, is now widely used to treat a wide range of conditions and was the fourth best-selling drug in the world in 2014 [25]. Daratumumab, designated for the treatment of multiple myeloma, has been approved seven times for different designations from 1983 to 2022 due to its unique mechanism of action [26]. 

The application of non-orphan drugs in the treatment of rare diseases is also frequently observed. By the end of 2017, 68 (14%) of the 489 orphan drugs designated in the United States were approved for non-orphan drug indications followed by orphan drug indications [8]. The above data fully demonstrates that cross-indication R&D and application of drugs is not an isolated event, but a scientific phenomenon with a certain universality and regularity. This is due to the high correlation of human physiological mechanisms and the commonality of disease pathogenesis. The cross-indication phenomenon that occurs during the R&D of drugs for target diseases is not only an in-depth exploration of the therapeutic potential of existing drugs, but also an important driving force to promote the innovation and development of the entire pharmaceutical field.

### Principle of affordability

Economism should be an important consideration in the allocation of resources, and this is particularly evident in the field of orphan drugs [27]. The total amount of public financial resources in different countries has a profound impact on the criteria and incentives for orphan drugs. The level of economic development, demographics, healthcare resources, and social values vary from country to country, making it difficult to transplant orphan drug designation criteria across countries. In countries with more abundant public financial resources, orphan drug-related systems are more easily implemented; whereas in countries with relatively tight public financial resources, there are trade-offs in financial expenditure, and the system of designating and incentivising orphan drugs is prone to controversy.

The system for designating orphan drugs should implement the principle of affordability and take into careful consideration the realities of the country’s economic construction and social development. Both overly loose and overly strict definitions of orphan drugs are not conducive to protecting patient rights [28]. Overly strict criteria can lead to insufficient drug development and market availability, leaving patients without treatment options. Overly loose criteria, on the other hand, can result in an excessive fiscal burden on the government, making it difficult to provide sustainable support for drug R&D. When the economic pressure greatly affects the accessibility and affordability of medical insurance for ordinary patients, the designation of orphan drugs and the incentive system will be hindered and difficult to implement. Therefore, for a country, it is necessary to adopt a two-pronged strategy: on the one hand, to build an effective legal system for orphan drugs to resolve the dilemmas of insufficient corporate motivation for drug R&D and poor drug accessibility, and to stimulate companies’ enthusiasm for engaging in orphan drugs R&D; on the other hand, to carefully review key data such as the disease spectrum and incidence rates of rare diseases in the country, and to take into account multiple factors, including the national socio-economic development situation and corporate R&D capabilities, in order to establish a scientific and rational orphan drug designation mechanism and other legal systems to safeguard the rights and interests of patients with rare diseases.

## The institutional construction of orphan drug designation in China

China’s orphan drug designation should be constructed and regulated in terms of the legislative model, the main body of the review, and the specific criteria.

### Legislative models

There are two main models of overseas legislation on the orphan drug designation: one is the “uniform legislation model”, and the other is the “uniform legislation+special provisions model”. Specifically, countries or regions such as Australia and the European Union have adopted the “uniform legislation model”, which stipulates the orphan drug designation system in the unified pharmaceutical law. In 1997, Australia amended the Therapeutic Goods Act 1990 to clarify the legal status of orphan drug designation [29]. In 2000, the European Union issued Regulation (EC) No 141/2000, mandatorily implementing a unified orphan drug designation system across its member states [30]. However, as global understanding of rare diseases deepens and patients’ unmet treatment needs grow, the EU has rolled out broad pharmaceutical regulatory legislative reforms in recent years, with the relevant bill now at the final legislative stage. On 26 April 2023, the European Commission (EC) tabled the legislative proposal COM(2023) 193 final, which repeals multiple previously existing pharmaceutical regulations including Regulation (EC) No 141/2000, and integrates all relevant pharmaceutical legislation into a new, more unified EU pharmaceutical regulatory framework. This is essentially an optimized upgrade of the “uniform legislation model” and does not alter its original legislative trajectory.The United States, Japan, and South Korea have adopted the “uniform legislation + special provisions model”. The United States enacted the Orphan Drug Act in 1983, the world’s first legislation on orphan drugs, which established the designation of orphan drug. Then in 1992, the United States issued the Orphan Drug Regulation, which detailed the authorities, procedures, and other aspects of orphan drug designation. In 1993, Japan revised the Pharmaceutical Affairs Law and the Rare Disease Pharmaceutical Management System, which respectively provide for the principle and detailed regulations on the designation of orphan drug. In 2016, South Korea enacted the Rare Disease Management Act, which defines concepts related to rare diseases and orphan drugs. In the same year, the Enforcement Rules of the Rare Disease Management Act was issued to further specify the designation criteria and management measures for rare diseases [31]. 

Comparing the characteristics of the two legislative models, the “uniform legislation model” facilitates the systematic and comprehensive regulation of various systems related to orphan drugs. It provides clear and consistent guidelines for multiple stakeholders, including enterprises, regulatory authorities, and patients, and enhances the operability and user-friendliness of the system. However, there are obvious shortcomings in the “uniform legislation model”. Unified legislation is often of a high legal rank, which makes it difficult to enact, stable but inflexible. In the current context of rapid advancements in life science and technology, which have injected strong innovative vitality into the field of orphan drugs, there is an urgent need for the system to respond comprehensively and promptly. Revisions and improvements to the system are inevitable. However, the optimization and adjustment of high-ranking legislation often require going through cumbersome legislative procedures, which can easily lead to delays in system optimization and an inability to meet the actual needs in a timely manner. The “uniform legislation+special provisions model” combines flexibility and efficiency. Uniform legislation establishes a basic framework for the orphan drugs system, and sets out general principles and rules, ensuring the system’s wholeness and consistency. Meanwhile, the special provisions address specific situations and emerging issues, refining the regulations based on the unified legislation. This model enables rapid responses and the formulation of corresponding rules according to the specific circumstances of different periods and fields.

In view of the fact that China is still in the early stages of building a legal system for orphan drugs, the “uniform legislation+special provisions model” is particularly suitable. China has already taken important steps in orphan drug legislation. Both the Pharmaceutical Administration Law of the People’s Republic of China and the Law of the People’s Republic of China on Basic Medical and Health Care and the Promotion of Health have made principled provisions for the research and marketing of orphan drugs, which have set the direction for the development of pharmaceuticals. On this basis, it is feasible to further clarify the designation system for orphan drugs through departmental regulations or even normative documents. Departmental regulations are characterised by relatively simple formulation procedures and high flexibility, which enables them to quickly respond to the practical needs of orphan drug system construction. In the initial stage, even normative documents can play an important role. Normative documents can promptly regulate specific issues in practice, fill institutional gaps, and once the relevant provisions become mature and stable, they can be elevated to regulations through legislative procedures to enhance their enforceability and stability.

### Designated institutions

Given the complexity and diversity of orphan drugs, and the fact that their designation serves as a unique pre-market procedure, most countries and regions that have enacted orphan drug legislation have established specialized review bodies to more accurately and efficiently carry out the designation work. For example, the U.S. FDA has an Office of Orphan Products Development (OOPD) [26]. Since 2000, COMP has been established under the European Medicines Agency (EMA) [32]. One of its statutory responsibilities is to conduct scientific assessments of orphan drug designation applications. The EC issues the final orphan drug designation decision based on the professional opinions provided by COMP. The legislative proposal COM(2023) 193 final, aimed at streamlining review and approval procedures and improving operational efficiency, will transfer regular decision-making authority for orphan drug designation from the EC to the EMA, revoke the statutory status of the EMA’s former independent standing Committee for COMP, integrate all COMP mandates into the Committee for Medicinal Products for Human Use (CHMP) [33]. In Japan, the Ministry of Health, Labour and Welfare (MHLW) carries out the designation process, in which the Pharmaceutical Affairs and Food Sanitation Council (PAFSC) under the MHLW first gives its opinion on the designation, and then the Minister of the MHLW takes into account the opinion of the PAFSC to make the final designation [34]. China has implemented a centralised and unified management system for medicines, with National Medical Products Administration(NMPA) being fully and centrally responsible for R&D, marketing and post-sale supervision of medicines. The designation of orphan drugs essentially falls within the scope of pharmaceutical management and is the prerequisite and foundation for the incentive mechanisms and approval for marketing of orphan drugs. Therefore, it should be exercised by the State Drug Administration. Specifically, drawing on the practices of the U.S. OOPD and the European Medicines Agency’s COMP, a Rare Disease Drugs Office could be established within the NMPA to address the unique characteristics of orphan drugs, such as low incidence rates, high R&D difficulties, and distinctive designation and incentive measures.

### Designation criteria

The designation criteria are the core of orphan drug designation. The current criteria for orphan drug designation outside of China mainly include economic criteria and medical criteria.

#### Representative legislative examples of the criteria for orphan drug designation

This section aims to study the representative legislative examples of orphan drug designation criteria in four countries or regions, namely the United States, the European Union, Japan and South Korea, in order to provide reference for the establishment of orphan drug designation criteria in China.

The United States and the European Union are among the countries or regions in the world that have adopted legislation to establish the criteria for orphan drug designation earlier, and where the designation has been applied more often in practice. Among them, the United States has the most lenient criteria and the earliest legislation [35]. There are two criteria in the United States, one of which can be met, including the epidemiological criterion, that is, the number of people targeted for treatment is less than 200,000; and the expected return criterion, i.e., although the number of people targeted for treatment is more than 200,000, R&D costs are difficult to be recovered within a specific period of time, which results in R&D costs being higher than the expected benefits of the drugs [36]. The EU orphan drug designation criteria include four criteria: critical illness, epidemiology, expected return and significant benefit [22]. The EU epidemiological criterion requires that the prevalence of the target rare disease does not exceed 5 per 10,000 of the total number of the EU, and by this definition, rare diseases may affect up to 30 million EU citizens [37]; the expected return criterion requires that the medicine has a low rate of return in the first ten years after marketing and is unlikely to generate sufficient economic return; [38] the critical illness criterion requires that the medicine is intended for the prevention, treatment and management of life-threatening or chronic wasting diseases, such as malignant neoplasms; The criterion of significant benefit means that the drug either fills the gap of the existing treatment or has more significant benefit compared with the existing treatment [39]. With the rapid development of modern medicine, some rare diseases have been alleviated to a greater extent, patients can be normal under standardised treatment, and should not become the object of national policy tilt care. Therefore, the criteria of critical illnesses and significant benefit are conducive to the concentration of national medical resources to meet the needs of people with key critical illnesses. Three core revisions to orphan drug designation criteria in the COM(2023) 193 final proposal: repealing the expected return criterion, long deemed to have no practical regulatory value; raising the “significant benefit” threshold, under which applicants must prove either no satisfactory authorised therapy for the target indication exists in the EU, or the candidate delivers verifiable clinical benefit over existing therapies; adding flexible epidemiological rules, where tailored prevalence thresholds for special diseases may be set per EMA advice, waiving the original mandatory ≤ 5/10,000 uniform threshold [33]. Japan and the Republic of Korea are two representative countries in Asia in terms of legislation on orphan drugs. Japan’s designation of orphan drugs includes three criteria, namely epidemiology, significant benefit and R&D feasibility which must be met simultaneously: the epidemiological criterion requires that the number of patients to be treated is less than 50,000; the significant benefit requirement is the same as that of the European Union; and the R&D feasibility criterion requires that the medicine has a theoretical basis for the treatment of the disease and that R&D programme is appropriate [37]. The R&D feasibility criterion was first proposed in Japan [40]. This additional requirement should have made it more difficult to obtain orphan drugs status in Japan, but the pass rate and approval rate for orphan drugs in Japan are higher than in the United States [41]. The main reason for this is that enterprises often apply for orphan drug designation in Japan for drugs that have already received orphan drug designation or approval in other countries [42]. This criterion has facilitated the rapid development of orphan drugs in Japan, but also led to the problem of Japan’s dependence on other countries for orphan drugs innovation and insufficient drug innovation [40]. In Korea, there are two main criteria [37]: epidemiological and significant benefit criteria. The epidemiological criterion means the target patients are less than 20,000; the significant benefit criterion is the same as those in the EU and Japan. However, for orphan drugs under development (in clinical trials, or the possibility of entering clinical trials has been confirmed), in addition to the above criteria, proof of the R&D feasibility is also required [43]. 

In summary, the epidemiological criterion is the core consideration in the designation of orphan drugs, and all other criteria are complementary to the epidemiological criterion, either for the purpose of expanding the scope of the designation to allow more drugs to enjoy the incentive mechanism, as in the United States, or for the purpose of pooling medical resources to enhance the quality of medical resource utilisation, as in Japan and other countries. The establishment of epidemiological criterion relies on the definition of the scope of rare diseases in a country. There is no internationally recognised definition of rare diseases, which must take into full consideration the country’s demographic and economic characteristics, level of medicine and medical needs. When the U.S. Orphan Drug Act was enacted in 1983, the incidence rate of 100,000 patients was used as the designation criterion, however, some scholars have proposed that this criterion leads to at least three diseases Tourette’s Syndrome, Multiple Sclerosis and Narcolepsy, which are excluded from the threshold, and the 1984 Orphan Drug Act was revised to formally adopt the 200,000 patients definition of rare diseases and pharmaceutical criterion [44]. More patients are included in the protection system of the rare disease system. In Korea, the Rare Disease Management Act defines rare diseases as those with fewer than 20,000 patients, or those that are difficult to diagnose and whose populations are unknown, and adopts a “catalogue management” approach to rare diseases [45]. 

The significant benefit criterion is stipulated by the European Union, Japan and South Korea, and occupies an important position in the designation of orphan drugs. The significant benefit criterion focuses on the effectiveness of drugs and reflects the basic demands of the orphan drugs protection system. The establishment of this criterion is not only conducive to the maintenance of the legal status and benefits of the original drug enterprises, but also helps to enhance the motivation of the enterprises for new drug R&D, and promotes the continuous innovation and development of the orphan drugs industry.

In addition to this, the United States and the European Union have stipulated the expected return criterion, which focuses on the cost of drug development and the rate of return of the drug over the period of time. Japan and South Korea (for orphan drugs under development) have stipulated the R&D feasibility criterion, which emphasises the reasonableness and efficiency of the drug development plan, and restricts drug R&D, which is conducive to the centralised and efficient allocation of resources. Therefore, the proportion of orphan drugs in Japan that pass marketing approval and obtain designation is as high as 64.3% [46]. The main reason is that the orphan drug designation criteria in Japan and South Korea were established relatively late, and companies often rely on orphan drug designation data from the United States and the European Union for their own designations. The ratio of orphan drugs approval and designation in the US is 15.4% higher than the EU’s 7% because the US orphan drug legislation was enacted 17 years earlier than the EU’s, during which time the US has designated and approved more orphan drugs [46]. 

#### Establishing orphan drug designation criteria in China

While drawing on international experiences and systems, it is essential to confront the reality that China’s orphan drug R&D, as well as its relevant systems, are still in the early stages. Therefore, criteria should be established based on the principle of affordability, taking into account various objective factors in China, such as the number of patients with rare diseases and the country’s economic level:

Firstly, the expected return criterion is not applicable in China. This criterion, which considers the cost-benefit ratio of a drug, includes medicines with low rates of return in the category of orphan drugs and may even encompass drugs that do not treat rare diseases. Although other countries have established cost-benefit indicators, they are rarely used. Literature shows that in the EU, only one out of five products that met the cost-benefit criterion and applied for orphan drug designation was actually designated (and this designation was later withdrawn by the applicant) [47]. Moreover, in the process of drug commercialization, the returns on drugs after market entry are highly uncertain and influenced by a range of complex factors, such as pharmaceutical policies, taxation, drug pricing, and health insurance issues, making it difficult to determine the actual returns on marketed drugs.

Secondly, the criterion of critical illness is not recommended to be applied. The criterion of critical illness is stipulated by the European Union, with the main purpose of concentrating medical resources and giving priority to the protection of the rights and interests of people with critical illnesses. Given that China’s rare disease drug system is in its infancy, the hasty application of this criterion may lead to an imbalance and weakening of the attention of the rare disease community. Moreover, China currently relies on the “Rare Disease Catalogue” to define the scope of rare diseases and to determine the scope of orphan drugs. The National Health Commission, in conjunction with relevant departments, jointly released two batches of rare disease catalogues in May 2018 and June 2023: the “First Batch of Rare Disease Catalogue” in 2018, which includes 121 rare diseases; and the “Second Batch of Rare Disease Catalogue” in 2023, which includes 86 rare diseases. The two catalogues together cover 207 rare diseases. Incorporating critical rare diseases into the rare disease catalogues can effectively replace the critical illness criterion, while also ensuring the efficiency of resource allocation.

Finally, it is recommended that China combine the three criteria of epidemiology, significant benefit and R&D possibilities, as described below:

##### First, epidemiological criterion

The epidemiological criterion is closely related to the definition of rare diseases in a country. Some countries directly define rare diseases based on the number of patients, such as the United States, Japan, and South Korea, while some regions define them based on the prevalence rate, such as the European Union. Most countries or regions in the world tend to define their rare diseases through legislation, such as the United States, Japan and the European Union, while some countries adopt a “legislative definition + catalogue listing” approach, such as South Korea. In terms of content, when setting epidemiological criterion, countries tend to take into account their national economic development, epidemiological characteristics, principles of protection and other factors, and it is difficult to form the uniform criteria worldwide in the short term.

In terms of legislative form, China can draw on the experience of South Korea by adopting a regulatory approach that combines “legislative definition” with “catalogue listing”. Based on the “Rare Disease Catalogue”, priority protection can be given to rare diseases with high incidence rates, severe impacts on children’s growth and development, and life-threatening conditions. The catalogue can enhance the efficiency of resource allocation. On the one hand, the definition of rare diseases forms the basis for selecting diseases for the catalogue, increasing the scientific and accurate nature of the selection process; on the other hand, it is conducive to gradually expanding the scope of rare diseases and orphan drugs. However, the current issue is that China does not have a formal, legal definition of rare diseases. In 2021, a multidisciplinary expert seminar organised by the Joint Meeting of Chairpersons of National Rare Disease Academic Groups and other organisations released the “Research Report on the Definition of Rare Diseases in China”, which defines rare diseases as “those with a neonatal incidence rate of less than 1 per 10,000, a prevalence rate of less than 1 per 10,000, and a number of people suffering from the disease of less than 140,000” [48]. The criteria define rare diseases not only by incidence and prevalence, but also by the number of patients, including not only the incidence of rare diseases in newborns, but also the prevalence of adults and the number of patients, covering the whole life cycle of patients’ care. The definition of adult prevalence rate as 1 per 10,000 is based on the characteristics of rare diseases included in the Catalogue of Rare Diseases, which contains 111 diseases that meet the epidemiological criterion of prevalence rate of less than 1 per 10,000, covering the most typical rare diseases. In the future, China should continue to improve the definition of rare diseases on the basis of existing research, and apply the definition of rare diseases in combination with the catalogue of rare diseases, so as to provide a clear basis for the designation of orphan drugs.

##### Second, significant benefit criterion

The criterion of significant benefit encourages the development of innovative drugs. Whether it is the innovation of diagnostic technology or the breakthrough of therapeutic methods, both can bring unprecedented hope to patients with rare diseases. Significant benefit also encourages medicines that offer superior value compared to existing therapies, which can be reflected in one or more aspects such as therapeutic effect, safety, or pricing. In terms of therapeutic effect, a new drug may be more effective in alleviating patients’ symptoms, significantly delaying disease progression, and even achieving disease reversal. For example, in some rare diseases, targeted therapies, compared to traditional chemotherapy drugs, can more precisely act on diseased cells. While improving therapeutic efficacy, they reduce damage to normal cells. Regarding safety, new drugs should have a lower incidence rate of adverse reactions or milder symptoms of adverse reactions, thereby enhancing patients’ medication adherence and quality of life. In terms of pricing, new drugs, through reasonable pricing and support from payment systems such as health insurance, can significantly reduce the economic burden on patients, which can also be considered as having superior value.

##### Third, research and development feasibility criterion

The R&D feasibility criterion puts forward higher requirements for the designation of orphan drugs, requiring that drug R&D plans be scientific and reasonable, with high economic feasibility, and that limited resources be effectively invested in drugs with high R&D potential and market value. The criterion of R&D feasibility is a double-edged sword: on the one hand, the criterion helps the incentives to be applied accurately to drugs with R&D potential; on the other hand, however, it may cause the applicant to rely on foreign orphan drug data, causing delays in the domestic launch of orphan drugs, and it may also lead to potentially valuable drugs being unable to advance further in R&D due to a lack of corporate funding [43]. 

Currently, orphan drug R&D in China is still in its infancy and mainly relies on imports. Establishing R&D feasibility criterion is conducive to the rapid designation and marketing of drugs that have already been designated in other countries. However, it is necessary to face the reality that this practice may lead to slow innovation of rare disease drugs in China and dependence on foreign data. On the one hand, the orphan drug system and industry development in foreign countries and regions such as the United States and the European Union are indeed earlier than China and relatively mature, and their beneficial experience can be used for our reference. On the other hand, by establishing systems such as orphan drug designation, we can continuously improve China’s orphan drug system and accelerate the rapid rise of China’s orphan drug industry.

## Conclusion

Currently, orphan drugs in China rely on the “Rare Disease Catalogue” for definition, and a systematic, comprehensive and scientific designation system has not yet been established. Establishing such a mechanism is imperative. The system will serve as the core driving force to promote R&D of orphan drugs and accelerate the launch of medicines.

Only by obtaining the designation for orphan drugs can enterprises enjoy various incentive measures from the government to support drug R&D. The system is also a powerful tool to prevent enterprises from abusing incentive measures. By clarifying the designation criteria, it is possible to effectively identify orphan drugs with R&D potential, prevent enterprises from abusing incentive measures to implement irrational R&D behaviors, and ensure the precise use of limited medical resources. From the progressive logic of institutional development, resolving the prerequisite issue of the orphan drug designation not only provides clear eligibility judgment criteria for our follow-up extended studies focusing on the localization of supporting incentive policies, but also lays a core foundation for building a full-chain regulatory system for rare disease medicinal products, which is of critical supporting value for promoting the continuous improvement of China’s regulatory system for rare disease medicines.Therefore, taking the orphan drug designation system as an entry point to build a comprehensive, systematic and efficient drug governance system for rare diseases in China is of great practical significance for promoting the innovative development of China’s pharmaceutical industry, enhancing the in-depth resource allocation and industrial transformation in the pharmaceutical field, and advancing the development of China’s rare disease cause.

## Data Availability

Not applicable.

## Abbreviations

WHO: World Health Organization
R&D: Research and Development
COMP: Committee for Orphan Medicinal Products
FDA: Food and Drug Administration
XLH: X-linked hypophosphatemia
ATMPs: Advanced Therapy Medicinal Products
OOPD: Office of Orphan Products Development
MHLW: Ministry of Health, Labour and Welfare
EC: European Commission
PAFSC: Pharmaceutical Affairs and Food Sanitation Council
EMA: European Medicines Agency
CHMP: Committee for Medicinal Products for Human Use
NMPA: National Medical Products Administration
NORD: National Organization for Rare Disorders
EURORDIS: European Organisation for Rare Diseases
